# Rehabilitation effect of intelligent rehabilitation training system on hemiplegic limb spasms after stroke

**DOI:** 10.1515/biol-2022-0724

**Published:** 2023-09-30

**Authors:** Mingqing Hao, Qian Fang, Bei Wu, Lin Liu, Huan Tang, Fang Tian, Lihua Chen, Demiao Kong, Juan Li

**Affiliations:** Nursing Department, Guizhou Provincial People’s Hospital, Guiyang 550000, Guizhou, China; College of Nursing, Guizhou University of Traditional Chinese Medicine, Guiyang 550000, Guizhou, China; Rory Meyers School of Nursing, New York University, New York 10012, New York, USA; College of Nursing, Zunyi Medical University, Zunyi 563000, Guizhou, China

**Keywords:** hemiplegic limb spasms, intelligent RTS, rehabilitation efficacy, stroke patients

## Abstract

This article aimed to explore the rehabilitation efficacy of intelligent rehabilitation training systems in hemiplegic limb spasms after stroke and provided more theoretical basis for the application of intelligent rehabilitation systems in the rehabilitation of hemiplegic limb spasms after stroke. To explore the rehabilitation efficacy of intelligent rehabilitation training system (RTS for short here) in post-stroke hemiplegic limb spasms, this study selected 99 patients with post-stroke hemiplegic limb spasms admitted to a local tertiary hospital from March 2021 to March 2023 as the research subjects. This article used blind selection to randomly divide them into three groups: control group 1, control group 2, and study group, with 33 patients in each group. Control group 1 used a conventional RTS, group 2 used the brain–computer interface RTS from reference 9, and research group used the intelligent RTS from this article. This article compared the degree of spasticity, balance ability score, motor function score, and daily living activity score of three groups of patients after 10 weeks of treatment. After 10 weeks of treatment, the number of patients in the study group with no spasms at level 0 (24) was significantly higher than the number of patients in group 1 (7) and group 2 (10), with a statistically significant difference (*P* < 0.05); In the comparison of Barthel index scores, after ten weeks of treatment, the total number of people in the study group with scores starting at 71–80 and 81–100 was 23. The total number of people in the score range of 71–80 and 81–100 in group 1 was 5, while in group 2, the total number of people in this score range was 8. The study group scored considerably higher than the control group and the difference was found to be statistically relevant (*P* < 0.05). In the Berg balance assessment scale and motor function assessment scale, after 10 weeks of treatment, the scores of the study group patients on both scales were significantly higher than those of group 1 and group 2 (*P* < 0.05). The intelligent RTS is beneficial for promoting the improvement of spasticity in stroke patients with hemiplegic limb spasms, as well as improving their balance ability, motor ability, and daily life activities. Its rehabilitation effect is good.

## 1 Introduction

Stroke is a collective term for a type of acute cerebrovascular disease, which seriously threatens the health and life of older and middle-aged persons [1]. It is characterized by high incidence rate, high disability rate, high mortality, high recurrence rate, and high economic burden. Due to the advancement of medical technology and the development of medical information services, the mortality rate of stroke has decreased, but patients often experience hemiplegia and limb spasms after stroke. Spasm is a chronic motor disorder caused by damage to upper motor neurons. Its clinical manifestation is characterized by an increase in muscle tension characterized by a velocity-dependent enhancement of the stretching reflex, accompanied by an active tendon reflex, which is caused by an increase in the excitability of the stretching reflex. About 65% of patients after stroke would experience spasms, and appropriate spasms are beneficial for patients, while excessive spasms can affect their motor function and daily activities, which is not conducive to their prognosis [2]. Therefore, effective rehabilitation training for patients with hemiplegic limb spasms after stroke is of great practical significance. However, at present, conventional rehabilitation training mainly relies on physical methods, medication methods, orthosis methods, and nerve block methods, which have poor therapeutic effects and have not formed a systematic rehabilitation training system (RTS). This article combines the current popular artificial intelligence and computer technology to improve the rehabilitation effect of hemiplegic limb spasms in patients, indicating that the intelligent RTS can be applied to the rehabilitation training of hemiplegic limb spasms in stroke patients. This article aims to improve the rehabilitation efficacy of stroke hemiplegic limb spasms through the research of intelligent RTS and provide more theoretic foundation for the use of artificial intelligence and computer technology in the field of medical rehabilitation.

With the continuous improvement of current medical technology, research on the rehabilitation of hemiplegia after stroke has also attracted increasing attention [3]. Many scholars have explored rehabilitation training for stroke hemiplegia and proposed different methods to promote limb rehabilitation for patients. Kim studied the impact of morning walking-assisted gait training on the rehabilitation of stroke patients with hemiplegia. He demonstrated through controlled trials that combining morning exercise-assisted gait training with traditional physical therapy can improve the autonomy and balance of stroke hemiplegic patients, promoting their recovery [4]. Zhu et al. demonstrated the effectiveness of yin-yang balancing acupuncture in cooperation with rehabilitation training and single rehabilitation training in the treatment of upper limb spasticity in stroke patients with hemiplegia [5]. Shao et al. studied the effect of non-hemiplegic side strength training on balance function, activity ability, and muscle strength of stroke patients. He showed through randomized controlled trials that rehabilitation strength training based on the national medical service system can promote the balance, flexibility, and muscle strength recovery of the paralyzed side of stroke patients [6]. Tomida et al. research is a randomized controlled trial using gait-assisted robots for gait training in stroke patients with hemiplegia. He said the gait-motor-assisted robot can enhance the walking capability of subacute stroke patients, to optimize the difficulty of training and facilitate their physical rehabilitation [7]. These scholars’ research on rehabilitation training for hemiplegia after stroke has enriched their theoretical content, which provides more possibilities for the recovery of limb function in patients with hemiplegia, but there are also some shortcomings. Although scholars have different opinions on rehabilitation training for hemiplegia after stroke, there has never been a systematic RTS formed, nor has it been combined with current intelligent technologies. This leads to the lack of significant reference value for research.

Some scholars have also applied RTS to the rehabilitation of stroke patients and elaborated on the role of RTS. Kim et al. evaluated the impact of a novel, portable upper limb training method for chronic stroke patients. He demonstrated through experiments that the upper limb training system improved upper limb function and cognitive ability in stroke patients after 6 weeks of training [8]. Yang and Li have developed a brain–computer interface RTS for post-stroke patients. He stated that the system can accurately identify the patient’s movement intention in real time and then trigger the operation of rehabilitation peripheral devices to provide feedback from visual, auditory, and tactile perspectives. The active rehabilitation of patients is also achieved through this system [9]. Scholars’ research on RTS can provide some theoretical support for this article, but due to the lack of emphasis on exploring the rehabilitation effectiveness of RTS for hemiplegic limb spasms after stroke, this research cannot be well applied. Through the research of scholars, it can be seen that there is relatively little discussion on the rehabilitation efficacy of intelligent RTS in hemiplegic limb spasms after stroke, and more research is needed in the future.

To better promote the rehabilitation of hemiplegic limb spasms after stroke, this article optimizes the rehabilitation of hemiplegic limb spasms after stroke based on theoretical analysis of intelligent RTS and previous scholars’ exploration of hemiplegic limb spasms rehabilitation training. This article conducted empirical research on the efficacy of the proposed rehabilitation method and found that the intelligent RTS in this article can better promote the relief of spasms in patients. Compared with traditional RTS, the innovation of this system lies in its attention to the importance of artificial intelligence and computer technology and its application in the construction of RTS. This helps to improve the rehabilitation effectiveness of RTS for stroke hemiplegic limb spasms.

## 2 Rehabilitation methods for hemiplegic limb spasms after stroke

### 2.1 Rehabilitation of hemiplegic limb spasms after stroke

#### 2.1.1 Poststroke hemiplegic limb spasms

Spasm is a common symptom in stroke patients [10]. After stroke, due to the disorder of the central motor inhibitory system, the mutual restriction and influence of *α* and *γ* motor neurons are dysregulated, giving *γ* neurons an advantage. Excessive release of lower motor neuron function can cause muscle spasms or abnormal excitation, accompanied by weakness and low muscle tone in antagonistic muscles. This can lead to imbalanced muscle movement, which in turn can cause movement disorders in the limbs. The increase or spasm of muscle tone in hemiplegic limbs after stroke is an important link in the rehabilitation of hemiplegic limbs, while excessive spasm is an important factor affecting the recovery of motor function in hemiplegic limbs [11]. Normal muscle tone and strength are the basic conditions for exercise. After stroke, it can lead to various functional impairments, among which unilateral limb motor dysfunction and limited daily living activities are the most common. The main purpose of stroke rehabilitation is to maximize the recovery of patients’ motor ability, enhance their ability to engage in daily activities, and enable them to participate in social activities again.

#### 2.1.2 Rehabilitation training for hemiplegic limb spasms after stroke

Rehabilitation training is a necessary step for stroke patients with hemiplegia to receive effective treatment and recover [12]. The occurrence of spasms in patients can hinder the recovery of limb function, and normal muscle tone is an important prerequisite for the recovery of limb function [13]. In the early stages of the rehabilitation of stroke patients with hemiplegia, it is possible to increase their spastic symptoms [14]. It requires medical staff to selectively carry out anti-spastic treatment based on the actual situation of the patient.

Rehabilitation training can alleviate muscle spasms in stroke patients and improve their motor ability [15]. Although traditional rehabilitation training can provide targeted treatment and adjust the intensity of treatment in a timely manner according to the patient’s course, muscle tone, and muscle strength, it cannot guarantee the treatment intensity of the patient within a certain treatment period. For patients with severe limb spasms after stroke or limb spasms after stroke, conventional rehabilitation training-induced central nervous system compensation and functional reorganization are usually difficult to achieve the goal of relieving spasms. The intelligent RTS can reduce the tension of the extensor muscles of the limbs, enhance the strength of the quadriceps femoris muscle and cricoid muscles, and significantly improve the stability and balance of the knee joint.

### 2.2 Intelligent RTS

#### 2.2.1 Overview of intelligent RTS

At present, there are generally two directions for research on rehabilitation therapy: one is virtual information technology and the other is the assistance of machine rehabilitation equipment. Machine rehabilitation equipment utilizes its own rehabilitation training program to provide targeted training to patients, achieving physical recovery by completing prescribed training tasks. At the same time, most machine-assisted devices have online communication capabilities, allowing patients to have face-to-face communication with doctors and other patients, facilitating consultation and communication. Doctors can also monitor and provide real-time guidance to patients throughout the process, maximizing the efficiency of rehabilitation training. During the rehabilitation process of patients, doctors can adjust their training plans in a timely manner based on their rehabilitation status, which plays a significant role in responding to emergencies. Artificial intelligence and computer technology can be applied to rehabilitation training, which can improve the overall functionality of the RTS. When using the training system, patients can engage in targeted exercise according to their own needs to achieve the best exercise results.

#### 2.2.2 Design of intelligent RTS

Because when using rehabilitation machines for rehabilitation training of stroke hemiplegic limb spasms patients, there would be motion coupling and force interaction between the end of the machine and the patient’s arm. Patients and rehabilitation machines can be viewed as a system and subjected to relevant dynamic analysis. Human limbs are usually simplified as two connected series structures, which achieve rehabilitation training through force interaction with rehabilitation and rehabilitation.

To achieve dynamic analysis of rehabilitation machines, Lagrange equation is a commonly used analysis method. If Lagrange equation is directly used to analyze the motion trajectory of its system, it would become very complex. This is because the rehabilitation machine is a planar parallel structure with four joint angles interrelated, which makes it difficult to obtain the deviation formula for its connecting rod position and driving joint angle. In response to this situation, the rehabilitation machine should be divided into three linkage structures with two degrees of freedom, and each connected branch should be processed separately. Then, the dynamic equations of the rehabilitation machine training system should be derived:

(1) \[P\ddot{A}+L\dot{A}=H,]\]

(2) \[P={P}_{1}+{P}_{2}+{P}_{3},]\]

(3) \[L={L}_{1}+{L}_{2}+{L}_{3},]\]

(4) \[H={W}_{1}^{-S}({{\vartheta }}_{1},{\gamma }_{1}){\varepsilon }_{1}+{W}_{2}^{-S}({{\vartheta }}_{2},{\gamma }_{2}){\varepsilon }_{2}+{W}_{3}^{-S}({{\vartheta }}_{3},{\gamma }_{3}){\varepsilon }_{3}.]\]

Among them: *P* – inertia; *L* – centripetal force; *H* – equivalent force of joint torque at the end; \[{P}_{1},{P}_{2},{P}_{3},{L}_{1},{L}_{2},{L}_{3}]\] – inertia term and centripetal force term of three series branches; \[{W}_{1}^{-S},{W}_{2}^{-S},{W}_{3}^{-S}]\] – Jacobian matrix; and \[{\varepsilon }_{1},{\varepsilon }_{2},{\varepsilon }_{3}]\] – joint torque of three branches.

Rehabilitation machines interact through impedance control methods. The impedance of the rehabilitation machine is defined as the relationship between input force \[H]\] and motion speed \[\dot{A}]\] , and its impedance form can be expressed as:

(5) \[H=-M({A}_{(s)}-{A}_{0})-Z{\dot{A}}_{(s)}-F{\ddot{A}}_{(s)}.]\]

Among them: \[M,Z,F]\] – stiffness, damping, and inertia.

The relationship between the impedance of the rehabilitation machine in another formula is achieved by the joint torque of a planar parallel structure, which is:

(6) \[S={W}^{S}({\vartheta })H=-{W}^{S}({\vartheta }){[}M(Q({\vartheta })-{A}_{0})+Z(W({\vartheta })\dot{{\vartheta }})+F(W({\vartheta })\ddot{{\vartheta }}+W({\vartheta })\dot{{\vartheta }})].]\]

Among them: \[Q]\] – the principle of forward dynamics of rehabilitation machines and \[{\vartheta }]\] – joint angle.

**Figure 1:**
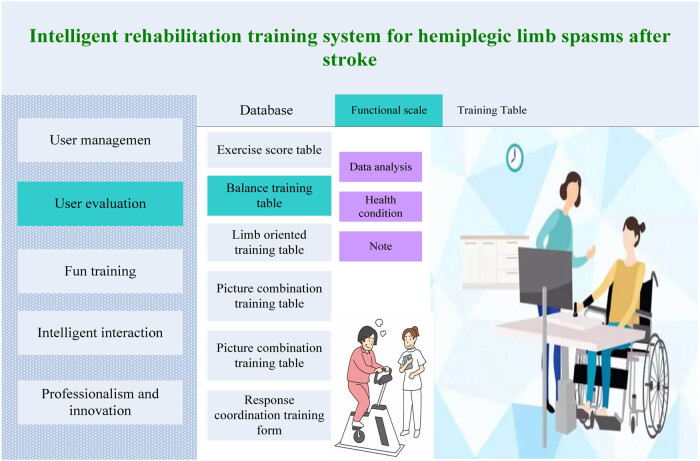
Interface diagram of the intelligent RTS for hemiplegic limb spasms after stroke.

The key point of the intelligent RTS in this article is to collect motion information provided by patients and rehabilitation machines. The intelligent RTS can collect data through the interaction between patients and rehabilitation machines and then integrate these two actions into a dataset. By comparing these two actions, a suitable training scenario and training tasks for the patient can be integrated and, then, sent to the client. It is transmitted from the client to the server for software evaluation, and the evaluated results are sent to the attending physician. Finally, the attending physician analyzes the system analysis results and provides final treatment suggestions and plans. The interface of the intelligent RTS for hemiplegic limb spasms after stroke is depicted in Figure 1:

The intelligent RTS for hemiplegic limb spasms after stroke in this article mainly consists of five parts, namely user management, user evaluation, fun rehabilitation training, intelligent interaction, and professional and innovative. The system uses a client server mode, which has the advantages of strong interactivity, fast client response speed, and low server workload. Patients need to undergo functional assessment before diagnosis and treatment. It is possible to determine whether a patient needs hospitalization for rehabilitation treatment based on patient information and evaluation results. After rehabilitation treatment is completed, it is also necessary to score the patient’s various functional conditions. Patients who meet the evaluation criteria are considered eligible to be discharged and go home for rest.

## 3 Empirical analysis of the rehabilitation effect of intelligent RTS on hemiplegic limb spasms after stroke

### 3.1 Materials and methods

#### 3.1.1 General information

This article selects 99 patients with hemiplegic limb spasms after stroke admitted to a local tertiary hospital from March 2021 to March 2023 as the research subjects. They are divided into three groups using the blind selection method, namely group 1, group 2, and the study group. There are 33 patients in each group. Control group 1 used a conventional RTS, group 2 used the brain–computer interface RTS from reference 9, and the research group used the intelligent RTS from this article. There was no statistically difference in basic data among the three groups of patients (*P* > 0.05), indicating comparability. The specific situation of the three groups of patients is depicted in Table 1.

**Table 1:** Comparison of general information among three groups of patients

General information	Group	Control group 1	Control group 2	Study group
Gender	Man	17	15	16
	Woman	16	18	17
Age		\[58.06\pm 7.82]\]	\[57.94\pm 8.02]\]	\[58.45\pm 7.55]\]
Course of disease		\[10.61\pm 2.75]\]	\[11.12\pm 2.38]\]	\[12.36\pm 2.37]\]
Disease type	Cerebral hemorrhage	15	14	14
	Cerebral infarction	18	19	19

Inclusion criteria are as follows: those who meet the clinical diagnostic criteria for hemiplegic limb spasms after stroke and patients without other sequelae of stroke. Exclusion criteria are as follows: patients with severe neurological disorders and patients with comorbidities such as heart, liver, and kidney. The patient is informed and the patient and their family members voluntarily sign an informed consent form.

#### 3.1.2 Methods

Control group 1 used a conventional RTS. Neurostimulation techniques can be used to passively exercise patients with physical therapists, keeping each movement in an anti spastic state for 2–4 min. Reflective suppression can be used by controlling the head and torso parts. This article combines some physical factor therapy methods, once a day for 45 min each time.

Control group 2 used a brain–computer interface RTS (method in ref. [9]). Brain–computer interface system refers to an interface device based on electroencephalogram (EEG) signals [16]. An EEG data collector can be worn on the patient’s head, allowing them to imagine movement and collect their EEG signals under the guidance of virtual reality animations. By wirelessly transmitting to the host, motor awareness can be recognized, functional electrical stimulation can be controlled, and electrodes attached to the limbs can be used to treat the patient’s limbs, thereby achieving the goal of assisting rehabilitation training. After entering the system, it guides patients to relax, calms them down, and then allows them to exercise, collect EEG data during relaxation, and record it. Treatment includes two types: repeated treatment and single treatment, with a treatment time of 20 min. During the treatment process, the doctor needs to guide the patient to follow the virtual reality images presented on the computer screen, allowing them to focus on imaginative actions. After successful imagination, the patient would receive functional electrical stimulation for treatment.

The research group adopts an intelligent RTS, which includes the upper rotation and the lower boarding wheel. According to the specific situation of the patient, three modes can be selected: passive training, assisted training, and active training. Corresponding game training can be conducted from the perspectives of physical rehabilitation, cognitive rehabilitation, and balance training. There are hard base eye-opening balance training, upper limb orientation training, reaction coordination training, and image combination training. The passive and active training methods alternate, and the resistance of the intelligent RTS can be adjusted in view of the patient’s condition. The speed is 15–30 rpm, once a day, for 20 min each time.

#### 3.1.3 Observation indicators

(1) The degree of spasms in patients can be compared before and after ten weeks of treatment. (2) It is possible to compare the better balance ability. (3) It can compare the motor function of three groups of patients before and after 10 weeks of treatment. (4) It can compare the daily living activities.

#### 3.1.4 Statistical methods

The data analysis was conducted using SPSS 17.0. The mean ± standard deviation (± SD) can be used to express the measurement data that conforms to the normal distribution. The *P* value (a parameter used to judge the hypothesis test results) is used between groups for grouping comparison. *P* < 0.05 means the variation is found to be statistically significant.

### 3.2 Result analysis

#### 3.2.1 Spasticity degree

**Figure 2:**
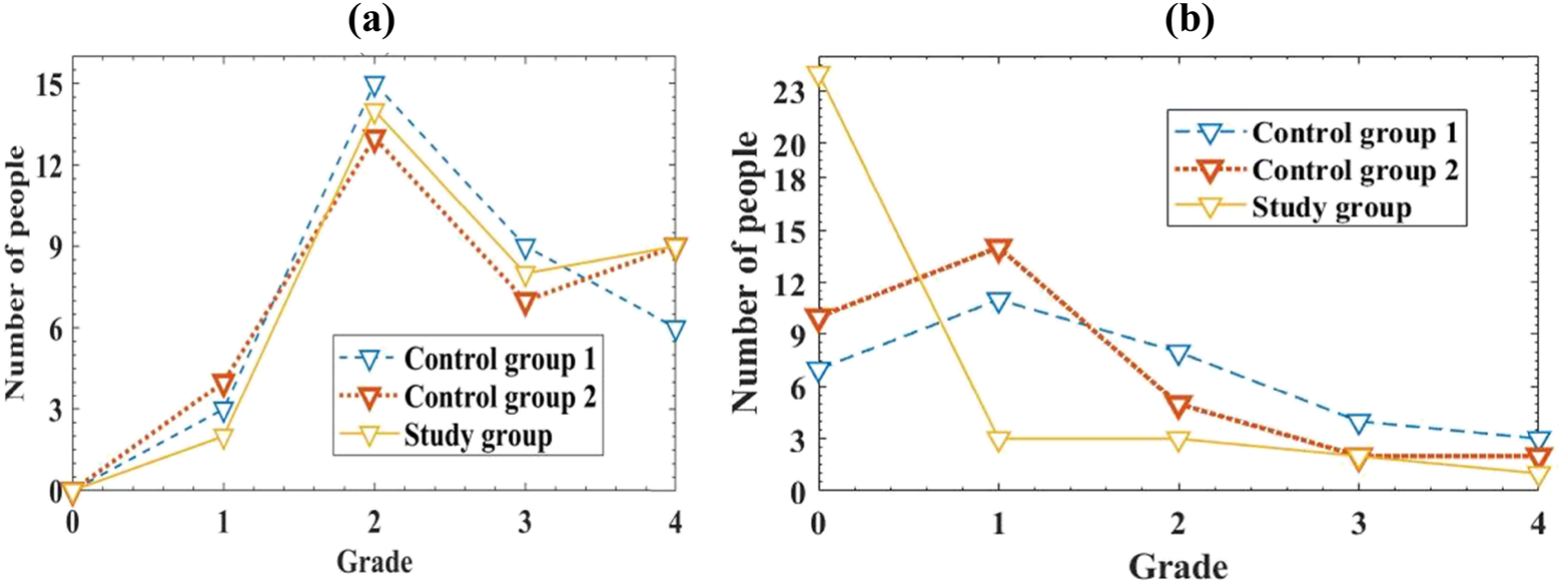
Comparison of spasticity levels. (a) Spasms before treatment and (b) spasms after treatment.

To compare the degree of spasticity among three groups of patients, this article evaluated the spasticity level of patients before and after treatment using the Modified Ashworth spasticity scale, which was divided into five levels. Level 0 indicates muscle relaxation without spasms; level 1 refers to mild spasms, where muscles experience slight resistance when rapidly stretched; level 2 refers to obvious spasms. When rapidly stretched, the muscles produce significant resistance, but can still stretch; level 3 is severe spasms. When rapidly stretched, the muscles have strong resistance and require many stretching movements to succeed; level 4 refers to extreme spasms, where muscles become stiff and unable to stretch. The spasticity grades of the three groups of patients under different methods before and after treatment are indicated in Figure 2.

The horizontal axis represents the level of spasms in the patient; the vertical axis represents the number of patients with different levels of spasms.

From Figure 2a, it can be seen that before treatment, there were 15 patients with grade 2 obvious spasms in group 1, and 13 patients with grade 2 obvious spasms in group 2. There were 14 patients in the study group with grade 2 obvious spasms. Overall, there was no discernible difference in spasticity among the three groups of patients before treatment (*P* > 0.05), and there was a certain degree of comparability in the study. From Figure 2b, it can be seen that after treatment, there were 7 patients in group 1 with no spasms at level 0 and 11 patients with mild spasms at level 1; there were 8 patients with grade 2 obvious spasms, 4 patients with grade 3 severe spasms, and 3 patients with grade 4 extreme spasms; in group 2, there were 10 patients with no spasms at level 0 and 14 patients with mild spasms at level 1; there were 5 patients with grade 2 obvious spasms, 2 patients with grade 3 severe spasms, and 2 patients with grade 4 extreme spasms; there were 24 patients in the study group who did not experience spasms at level 0, 3 patients with mild spasms at level 1, and 3 patients with significant spasms at level 2; there were 2 patients with grade 3 severe spasms, and 1 patient with grade 4 severe spasms. The number of patients in the study group who did not experience spasms at level 0 was significantly higher than that of group 1 and group 2, with a difference (*P* < 0.05). This indicates that the intelligent RTS in this article has a significant rehabilitation effect on hemiplegic limb spasms in stroke patients, which is more conducive to the rehabilitation of patients after spasms.

#### 3.2.2 Balance ability analysis

**Figure 3:**
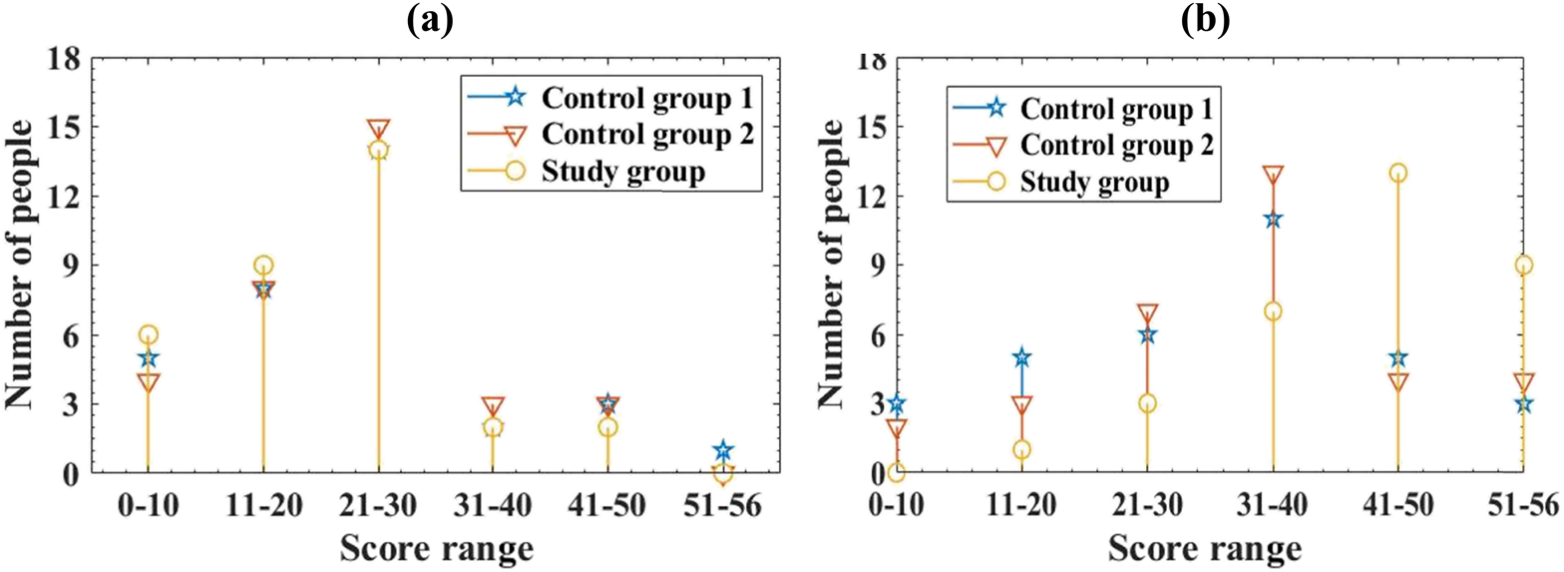
Comparison of Berg balance assessment scale scores. (a) Berg balance assessment scale scores before treatment and (b) Berg balance assessment scale scores after treatment.

This article uses the Berg Balance Scale to compare the balance ability of three groups of patients. This table has a total of 14 items, each with a score of 0–4, totaling 56 points. The higher the score, the better its balance ability. This article divides the score range of the scale into 0–10 points, 11–20 points, 21–30 points, 31–40 points, 41–50 points, and 51–56 points. This article summarizes the overall scores of the Berg balance assessment scale, as depicted in Figure 3.

The horizontal axis represents the score range of the scale, while the vertical axis represents the number of scoring individuals in different score ranges.

From Figure 3a, it can be seen that before treatment, the Berg balance assessment scale scores of the three groups of patients were mostly concentrated in the range of 21–30 points. Among them, 14 patients in group 1 scored in this range, while 15 patients in group 2 scored in this range. There were 14 patients in the study group who scored in this range, and there was no discernible difference in overall scores among the three groups of patients (*P* > 0.05). According to Figure 3b, after treatment, there were 3 individuals in group 1 with scores starting at 0–10, 5 individuals in the control group 11–20, 6 individuals in the control group 21–30, 11 individuals in the control group 31–40, 5 individuals in the control group 41–50, and 3 individuals in the control group 51–56; in group 2, there were 2 individuals with scores starting at 0–10, 3 individuals with scores starting at 11–20, 7 individuals with scores starting at 21–30, 13 individuals with scores starting at 31–40, 4 individuals with scores starting at 41–50, and 4 individuals with scores starting at 51–56; in the research group, there were 0 people with scores starting at 0–10, 1 person with scores starting at 11–20, 3 people with scores starting at 21–30, 7 people with scores starting at 31–40, 13 people with scores starting at 41–50, and 9 people with scores starting at 51–56. The number of patients in the study group with a score range of 41–50 was significantly higher than that of group 1 and group 2, with a statistically significant difference (*P* < 0.05). After three different rehabilitation training methods, the Berg balance assessment scale scores of all three groups of patients were improved, indicating a significant improvement in their balance ability. The study group of patients who underwent the intelligent RTS in this article scored higher on the Berg balance assessment scale, indicating that the method proposed in this article is more helpful in promoting the improvement of patients’ balance ability and achieving better rehabilitation outcomes. This is because the system in this article combines intelligence with rehabilitation training, which can not only assist patients in rehabilitation training and better recovery but also provide real-time understanding of patients’ physical conditions, reasonable rehabilitation suggestions, and targeted training.

#### 3.2.3 Sports functional analysis

**Figure 4:**
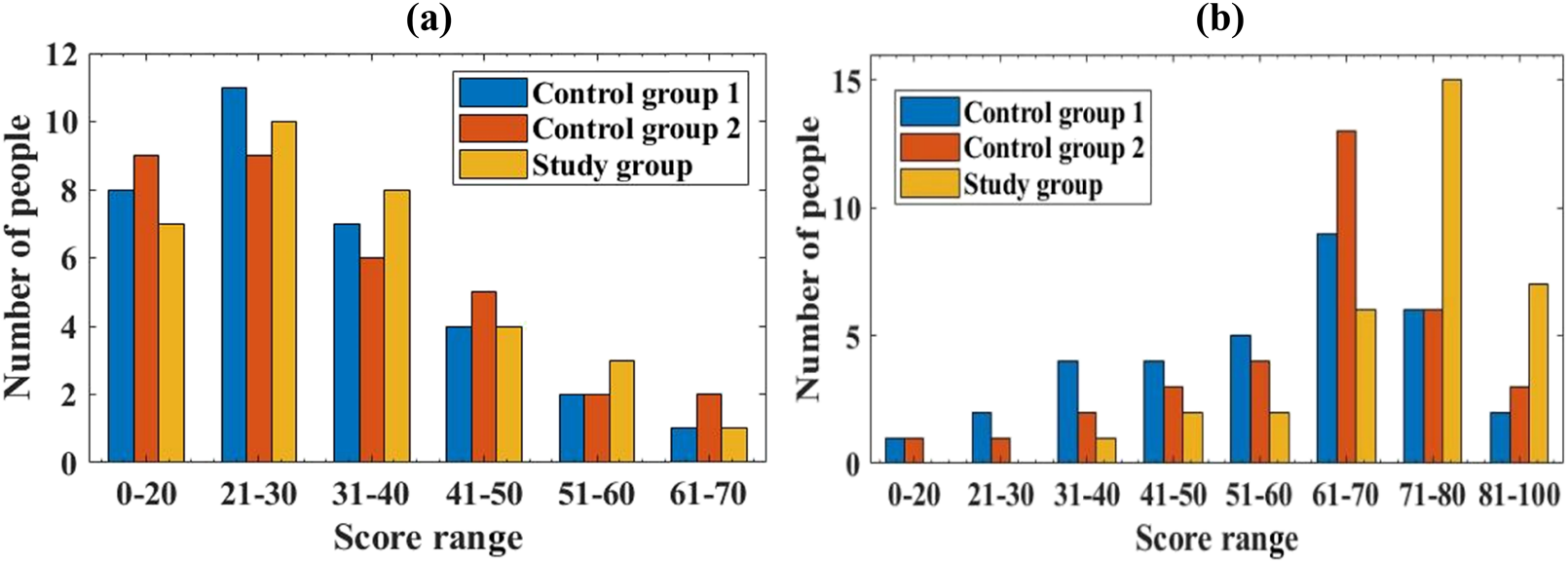
Comparison of overall scores on the motor function assessment scale. (a) Score of motor function assessment scale before treatment and (b) score of motor function assessment scale after treatment.

In addition to statistical analysis of the patient’s spasticity and balance ability, the patient’s exercise function was also analyzed. This article uses the simplified Fugl-Meyer assessment to compare the motor function. The scale has a total of 100 points, with a higher score indicating better motor function. This article divides the score range of the scale into 0–20 points, 21–30 points, 31–40 points, 41–50 points, 51–60 points, 61–70 points, 71–80 points, and 81–100 points. This article summarizes the overall scores of the motor function assessment scale, as depicted in Figure 4:

The horizontal axis represents the scale score range, while the vertical axis represents the number of patients in different score ranges. Due to the fact that the number of patients in the score range of 71–80 and 81–100 in Figure 4a is 0, it cannot be presented in the figure, so it is not included in Figure 4a.

From Figure 4a and b, it can be seen that there is no discernible difference in the number of patients in each score range before treatment (*P* > 0.05), and the study is comparative. After treatment, in group 1, there were 1 person with a score of 0–20, 2 people with a score of 21–30; there were 4 people with a score of 31–40, 4 people with a score of 41–50, and 5 people with a score of 51–60; there were 9 people with a score of 61–70, 6 people with a score of 71–80, and 2 people with a score of 81–100; in group 2, there were 1 person with a score of 0–20 and 1 person with a score of 21–30; there were 2 people with scores starting at 31–40 and 3 people with scores starting at 41–50; there were 4 people with scores starting at 51–60 and 13 people with scores starting at 61–70; there were 6 people with scores starting at 71–80 and 3 people with scores starting at 81–100; in the research group, there were 0 people with scores starting at 0–20 and 0 people with scores starting at 21–30; there were 1 person with a score of 31–40 and 2 people with a score of 41–50; there were 2 people with scores starting at 51–60 and 6 people with scores starting at 61–70; there were 15 people with scores starting at 71–80 and 7 people with scores starting at 81–100. It can be clearly seen that the number of people in the study group with scores starting at 71–80 and 81–100 is significantly higher than that of group 1 and group 2, with a statistically significant difference (*P* < 0.05). This indicates that the intelligent RTS in this article can improve the motor function assessment scale scores of the study group patients. This means that the motor function recovery of the study group patients is better than that of group 1 and group 2, indicating that the rehabilitation effect of the intelligent RTS is better, and the recovery effect of the patient’s motor function is more obvious.

#### 3.2.4 Daily life activity ability

**Figure 5:**
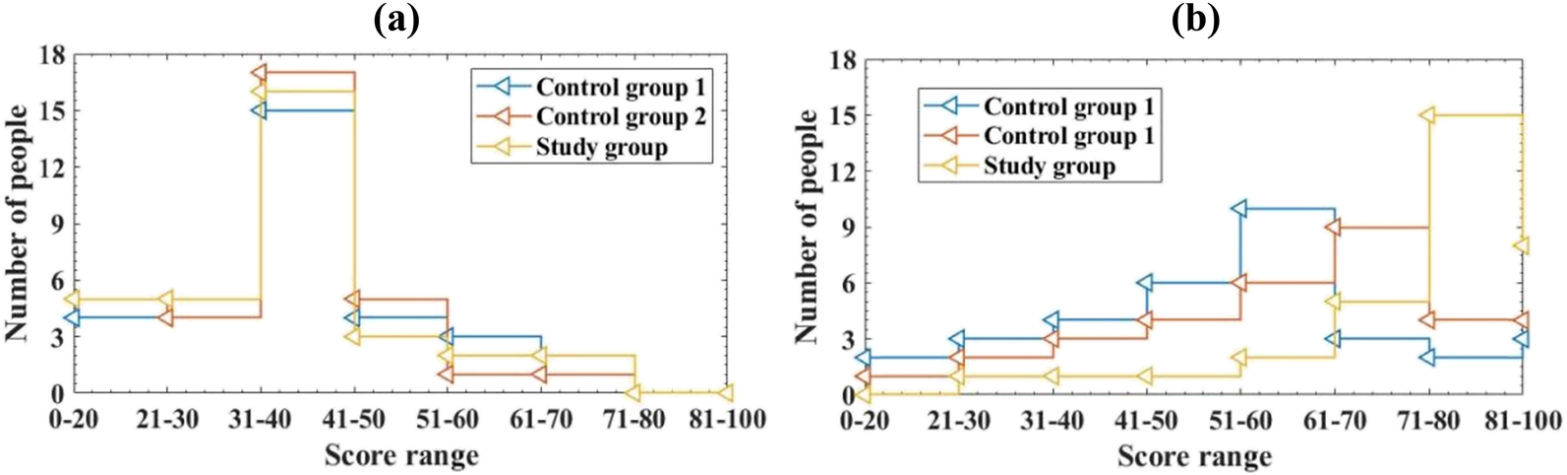
Comparison of Barthel Index scores. (a) The Barthel index evaluation table scores before treatment and (b) the Barthel index evaluation table scores after treatment.

Finally, the daily living activities of three groups of patients were compared. This article uses the modified Barthel index rating scale to compare the daily living activities of three groups of patients. The scale has a score of 100, and a higher score means that the patient’s daily living activities are better. Similarly, this article divides the score range of the evaluation table into 0–20 points, 21–30 points, 31–40 points, 41–50 points, 51–60 points, 61–70 points, 71–80 points, and 81–100 points. This article calculates the overall score of the Barthel Index assessment table, as depicted in Figure 5.

The horizontal axis represents the score range, and the vertical axis represents the number of patients within that score range.

From Figure 5a and b, it can be seen that there was no discernible difference in the Barthel index score among the three groups of patients before treatment (*P* > 0.05). After treatment, in group 1, there were 2 individuals with scores starting at 0–20, 3 individuals with scores starting at 21–30, and 4 individuals with scores starting at 31–40; there were 6 people from 41 to 50 and 10 people from 51 to 60; there were 3 people from 61 to 70 and 2 people from 71 to 80; there were three people from 81 to 100. In group 2, there were 1 person with a score range of 0–20 and 2 people with a score range of 21–30; there were 3 people from 31 to 40 and 4 people from 41–50; there were 6 people from 51 to 60 and 9 people from 61 to 70; there were 4 people from 71 to 80 and 4 people from 81 to 100; in the research group, there were 0 people with scores starting at 0–20 and 1 person with scores starting at 21–30; there were 1 person from 31 to 40 and 1 person from 41–50; there were 2 people from 51 to 60 and 5 people from 61 to 70; there were 15 people from 71 to 80 and 8 people from 81 to 100. After treatment, there were 23 patients in the study group with scores starting at 71–80 and 81–100, while there were 5 patients in group 1 with scores starting at 71–80 and 81–100. There were a total of 8 individuals in group 2 with scores starting at 71–80 and 81–100. It can be seen that the total number of patients in the study group with scores starting at 71–80 and 81–100 is larger than that in group 1 and group 2, with a difference (*P* < 0.05). This indicates that the method proposed in this article is more conducive to the recovery of patients’ daily living activities, and its rehabilitation effect is better.

In addition, this article counted the average scores of the Berg Balance Rating Scale, Motor Function Rating Scale, and Barthel Index Rating Scale, as depicted in Table 2.

**Table 2:** Average scores of scale tests after treatment

After treatment	Control group 1	Control group 2	Control group 3	*F*	*P*
Berg balance assessment scale	\[31.97\pm 12.34]\]	\[34.97\pm 12.30]\]	\[42.09\pm 9.39]\]	6.834	0.002
Motor function assessment scale	\[55.33\pm 16.61]\]	\[60.33\pm 15.86]\]	\[70.30\pm 12.62]\]	8.374	<0.001
Barthel index evaluation form	\[51.06\pm 17.31]\]	\[57.30\pm 17.09]\]	\[69.36\pm 12.99]\]	11.272	<0.001

According to Table 2, after 10 weeks of treatment, the average scores of the Berg balance assessment scale, motor function assessment scale, and Barthel index assessment scale in the study group were larger than those in group 1 and group 2. The *P*-values are all less than 0.05. This means that the rehabilitation efficacy of the intelligent RTS in this article is better than that of the conventional RTS and the brain–computer interface RTS in ref. [9].

### 3.3 Discussions

Rehabilitation training can effectively alleviate the symptoms of limb spasms and improve their limb motor ability. In long-term clinical work, it has been found that relying solely on traditional rehabilitation training cannot effectively alleviate hemiplegic limb spasms after stroke. The intelligent RTS formed by combining artificial intelligence and computer technology with rehabilitation training can not only ensure the targeted rehabilitation treatment, but also greatly reduce the workload of therapists. During the same training cycle, using an intelligent RTS can not only achieve high-intensity training for patients but also reduce the occurrence of limb spasms caused by upper motor neuron damage. This can improve the efficiency of rehabilitation training and is a promising new type of rehabilitation training method.

## 4 Conclusions

With the continuous promotion and application of artificial intelligence technology, artificial intelligence in the medical field has also achieved better development. However, there is still room for further exploration in introducing artificial intelligence into RTS. The theme of this article is the rehabilitation effect of an intelligent RTS on hemiplegic limb spasms after stroke. First, the relevant research background of this article was introduced, and then, the advantages and disadvantages of previous scholars’ research on stroke hemiplegia rehabilitation were summarized and analyzed. Then, based on the analysis of the current rehabilitation training for hemiplegia limb spasms after stroke, an intelligent RTS was introduced. To verify the effectiveness of the intelligent RTS, this article compared the rehabilitation efficacy of the system, conventional RTS, and reference 9 brain–computer interface RTS for hemiplegic limb spasms in stroke patients through a controlled experiment. The experimental results showed that after 10 weeks of treatment, the spasticity level of the study group patients who used the intelligent RTS as the rehabilitation method in this study was significantly higher among the three groups of patients. The balance ability, motor ability, and daily life activity ability were significantly superior to group 1 using conventional training systems as the rehabilitation method and group 2 using brain–computer interface RTS as the rehabilitation method, with statistically significant differences (*P* < 0.05). There are also some shortcomings in this study, and further improvement is needed. There are also some shortcomings in this study. Due to practical conditions, the overall number of samples selected in this study is relatively small, which may have a certain impact on the final results. In addition, as the intelligent RTS applied to hemiplegic limb spasms after stroke is a system that needs continuous improvement and optimization, the performance and operational requirements of the system would be higher and more user-friendly in the future, and further exploration is needed.
